# 

**Published:** 1906-06

**Authors:** 


*The following donations have been received since the publication of the List in March:*

*May 31st, 1906.*

| | |
|---|---|
| Albany Medical College (1) ... ... ... ... ... ... | 1 volume. |
| British Balneological and Climatological Society (2) | 1 ,, |
| The Editor of *Cleveland Medical Journal* (3) ... ... | 1 ,, |
| The Editor of *Detroit Medical Journal* (4) ... ... | 1 ,, |
| L. M. Griffiths (5) ... ... ... ... ... ... ... ... ... | 22 volumes. |
| W. Arbuthnot Lane (6) ... ... ... ... ... ... ... | 1 volume. |
| Luzerne County Medical Society (7) ... ... ... ... | 1 ,, |
| Providence Medical Association (8) ... ... ... ... | 1 ,, |
| R. Shingleton Smith, M.D. (9) ... ... ... ... ... ... | 2 volumes. |
| James Willing, Jun. Ltd. (10) ... ... ... ... ... ... | 1 volume. |

### SIXTIETH LIST OF BOOKS.

The titles of books mentioned in previous lists are not repeated.

The figures in brackets refer to the figures after the names of the donors, and show by whom the volumes were presented. The books to which no such figures are attached have either been bought from the Library Fund or received through the *Journal*.

**Bardswell, N. D.** *The Consumptive Working Man* ... ... ... ... ... ... 1906
**Battle and E. M. Corner, W. H.** *The Surgery of the Diseases of the Vermiform Appendix* ... ... ... ... ... ... ... 1904
**Bigg, H.** ... ... *Spinal Curvatures* ... ... ... ... ... ... ... ... ... ... 1905
**Blumfeld, J.** ... *Anæsthetics* ... ... ... ... ... ... ... ... ... 2nd Ed. 1906
**Broadbent, Sir W. H. and J. F. H.** *Heart Disease* ... ... ... 4th Ed. 1906
**Carson, H. W.** ... *Aids to Surgical Diagnosis* ... ... ... ... ... ... ... 1906
**Chaplin, E. H. Colbeck and A.** *The Science and Art of Prescribing* 2nd Ed. 1906
**Colbeck and A. Chaplin, E. H.** *The Science and Art of Prescribing* 2nd Ed. 1906
**Corner, W. H. Battle and E. M.** *The Surgery of the Diseases of the Vermiform Appendix* ... ... ... ... ... ... ... 1904
**Crowe, H. W.** ... *Consumption: Home Treatment and Rules for Living* ... 1906
**Cunningham, D. J.** [Ed.] *Text-Book of Anatomy*... ... ... ... 2nd Ed. 1906
**David, M.** ... ... *Grundriss der orthopädischen Chirurgie* Zweite Aufl. 1905
**Ehrlich, P.** [et al] *Diseases of the Blood.* (Translated Ed. by A. Stengel) 1905
**Emery, W. D'E.** *Clinical Bacteriology and Hæmatology* ... ... ... 1906
**Harrison, R.** ... *The Urethrotomies and Kidney Capsulotomy* ... ... ... 1906
**Herringham, W. P.** *On Physical Training in Schools* ... ... ... ... ... 1906
**Jaksch, R. von** ... *Clinical Diagnosis.* (Ed. by A. E. Garrod) 5th Ed. 1905
**Kirkbride, T. S.** ... *On Hospitals for the Insane* ... ... ... ... ... (5) 1854
**Lane, W. A.** ... *The Operative Treatment of Fractures* ... ... ... (6) 1905
**Lewis, J.** ... ... *Digest of the English Census of 1871* ... ... ... (5) 1873
**Loane, M.** ... ... *Simple Introductory Lessons in Midwifery* ... ... ... [N.D.]
**Lockwood, C. B.** ... *Appendicitis* ... ... ... ... ... ... ... ... 2nd Ed. 1906
**May and C. Worth, C. H.** *Diseases of the Eye* ... ... ... ... ... ... 1906
**Metchnikoff, E.** ... *Immunity in Infective Disease.* (Tr. by F. G. Binnie) 1905
**Monro, T. K.** ... *Manual of Medicine* ... ... ... ... ... ... 2nd Ed. 1906
**Noorden, C. von** ... *Diabetes Mellitus.* (Translated) .. ... ... ... ... 1906
**Osler, W.** ... ... *Aequanimitas, and other Addresses* ... ... ... ... 1905
**Pavy, F. W.** ... *Carbohydrate Metabolism and Diabetes* ... ... ... ... 1906
*Prevention of Disease, The.* (Tr. from the German) ... ... ... ... (9) 1902
**Shaw-Mackenzie, J. A.** *The Nature and Treatment of Cancer* ... 3rd Ed. 1906
**Thorburn, W.** ... *Course in Operative Surgery* ... ... ... ... ... ... 1906
**Tunstall, F. J. Warwick and A. C.** "*First Aid*" *to the Injured and Sick* 4th Ed. 1906
**Wallace, J. S.** ... *On Cause and Prevention of Dental Caries* ... ... ... 1906
**Walter, A. E.** ... *X-rays in General Practice* ... ... ... ... ... ... 1906
**Warwick and A. C. Tunstall, J. F.** "*First Aid*" *to the Injured and Sick.* 4th Ed. 1906
**Weber, Sir H.** ... *On Means for the Prolongation of Life* ... 2nd Ed. 1906
**Wellington, R. H.** *The King's Coroner.* Vol. II. ... ... ... ... ... 1906
**White, R. P.** ... *Catarrhal Fevers* ... ... ... ... ... ... ... ... 1906
**Wilks, S.** ... ... *Specific Fevers and Diseases of the Chest* ... ... (5) 1874
**Worth, C. H. May and C.** *Diseases of the Eye* ... ... ... ... ... ... 1906
**Young, A. H.** [Ed.] *Studies in Anatomy.* Vol. III. ... ... ... ... ... 1906

### TRANSACTIONS, REPORTS, JOURNALS, &c.

Albany Medical Annals ... ... ... ... ... ... ... ... (1) Vol. XXVI. 1905
American Journal of Medical Sciences, The ... ... ... Vol. CXXX. 1905
American Medicine ... ... ... ... ... ... ... ... ... ... Vol. X. 1905

| | | |
|---|---|---|
| American Ophthalmological Society, Transactions of the | Vol. X., Part 3 | 1905 |
| American Practitioner and News, The | Vol. XXXIX. | 1905 |
| American Society of Tropical Medicine, Papers read before the | Vol. I. | 1904-05 |
| American Surgical Association, Transactions of the | Vol. XXIII. | 1905 |
| Archives de Neurologie | Tom. XIX., XX. | 1905 |
| Archives of Pediatrics | Vol. XXII. | 1905 |
| Birmingham Medical Review, The | N.S., Vols. V., VI. | 1905 |
| Bookseller, The | | 1905 |
| Boston Medical and Surgical Journal, The | Vol. CLIII. | 1905 |
| Bristol Medico-Chirurgical Journal, The | Vol. XXIII. | 1905 |
| Bristol Port Sanitary District—Annual Report of the Medical Officers of Health for 1905 | | 1906 |
| Bulletin de l'Académie de Médecine | Tom. LIII., LIV. | 1905 |
| Canada Lancet, The | Vol. XXXVIII. | 1904-05 |
| Canadian Practitioner and Review, The | Vol. XXX. | 1905 |
| China Medical Missionary Journal, The | Vol. XIX. | 1905 |
| Cincinnati Lancet-Clinic, The | N.S., Vol. LV. | 1905 |
| Cleveland Medical Journal, The | (3) Vol. IV. | 1905 |
| Detroit Medical Journal | (4) Vol. V. | 1905 |
| Epidemiological Society of London, Transactions of the | Vols. XXI., 1902; XXIII., XXIV. | 1904-05 |
| Gazette de Gynécologie | Tom. XX. | 1905 |
| Gazette des Hôpitaux | 1904, | 1905 |
| Gazette hebdomadaire des Sciences médicales de Bordeaux | | 1905 |
| Hospitals—St. Bartholomew's Hospital Reports | Vol. XLI. | 1906 |
| Indian Medical Gazette, The | Vol. XL. | 1905 |
| Intercolonial Medical Journal of Australasia | Vol. X. | 1905 |
| Journal of Balneology and Climatology, The | (2) Vol. IX. | 1905 |
| Journal of Experimental Medicine, The | Vol. VII. | 1905 |
| Journal of Hygiene, The | Vol. V. | 1905 |
| Journal of Laryngology, The | Vol. XX. | 1905 |
| Journal of Nervous and Mental Disease, The | Vol. XXXII. | 1905 |
| Journal of Obstetrics and Gynæcology, The | Vol. VIII. | 1905 |
| Journal of the American Medical Association, The | Vol. XLV. | 1905 |
| Journal of the Royal Sanitary Institute | Vol. XXVI. | 1905 |
| Library, The | N.S., Vol. VI. | 1905 |
| Local Government Board, Supplement to 30th Annual Report (1900-01)—Lead Poisoning and Water Supplies | (9) | 1903 |
| Lunacy, 53rd Annual Report of the Commissioners in | (5) | 1899 |
| Luzerne County Medical Society, Transactions of the | (7) Vol. XIII. | 1905 |
| Medical Age, The | Vol. XXIII. | 1905 |
| Medical Annual, The | | 1906 |
| Medical News, The | Vol. LXXXVII. | 1905 |
| Medical Record | Vol. LXVIII. | 1905 |
| Medical Review, The | Vol. VIII. | 1905 |
| Medical Review of Reviews | Vol. XI. | 1905 |
| Medico-Chirurgical Transactions | Vol. LXXXVIII. | 1905 |
| Monthly Cyclopædia, The | N.S., Vol. VIII. | 1905 |

| | | |
|---|---|---|
| Münchener medicinische Wochenschrift | Bd. II. fur | 1905 |
| Nord médical, Le | Tom. XI. | 1905 |
| Northumberland and Durham Medical Journal, The | | 1905 |
| Odontological Society of Great Britain, Transactions of the | Vol. XXXVIII. | 1905 |
| Polyclinic, The | Vol. IX. | 1905 |
| Post-Graduate, The | Vol. XX. | 1905 |
| Practical Medicine | Vol. III. | 1905 |
| Practitioner, The | [Vol. LXXV.] | 1905 |
| Progrès médical, Le | Tom. XXI. | 1905 |
| Progressive Medicine | Vol. I. | 1906 |
| Providence Medical Journal, The | (8) Vol. VI. | 1905 |
| Revue de Thérapeutique | 1904, | 1905 |
| Revue générale d'Ophtalmologie | Tom. XXIV. | 1905 |
| Revue hebdomadaire de Laryngologie | Tom. XXV. | 1905 |
| St. Louis Medical Review | Vol. LII. | 1905 |
| St. Petersburger medicinische Wochenschrift | | 1905 |
| Sanitary Record, The | Vol. XXXVI. | 1905 |
| Therapeutic Gazette, The | Vol. XXIX. | 1905 |
| Travaux pratiques d'Obstétrique et de Gynécologie | | 1905 |
| Whitaker's Almanack | | (5) 1870-73 |
| Wiener klinische Wochenschrift | | 1905 |
| Willing's Press Guide | | (10) 1906 |
| Zentralblatt für Gynäkologie | | 1905 |
| Zentralblatt für innere Medicin | | 1905 |

### MEETINGS OF SOCIETIES.

### Bristol Medico-Chirurgical Society.

**University College, Bristol.**—Students of the College have been successful in the following recent Examinations:—

F.R.C.S.—*Primary Examination:* C. A. Moore, M.B., B.S. Lond.

Conjoint Board of England.—*First Examination: Chemistry and Physics*—J. W. Elliott. *Biology*—T. E. Ashley, L. C. W. Baker, H. H. Hiley, S. Marle, F. C. Nicholls. *Surgery*—W. W. King, R. E. Thomas, J. B. V. Watts*. *Midwifery*—F. T. Boucher, J. B. V. Watts*.

L.D.S.—*Chemistry and Physics*—P. J. Burton. *Dental Metallurgy*—C. A. Joll. *Final Examination: Part I.*—C. G. Plumley; *Part II.*—W. J. Jones*.

* Completes Examination.

Society of Apothecaries.—*Midwifery*—P. Moxey, E. V. Connellan.

**Orthopædic Hospital and Home for Crippled Children, Bristol.**—E. H. E. Stack, M.B., B.C. Cantab., F.R.C.S. Eng., has been appointed Honorary Surgeon to this institution.

**Bristol Eye Dispensary.**—The annual report of this institution shows that 5,056 patients have been treated during the year. The financial statement was satisfactory. It was stated that the examination of school children's eyes formed a considerable part of the work of the honorary staff, and it was suggested that the Education Committee should pay some medical man who could undertake such work.

**Winsley Sanatorium.**—The first annual report—that for 1904-5—has been issued, and is a well-illustrated record of everything relating to the institution. The report of the Medical Officer shows that excellent work is being done, the results for the year being as shown below:—

TABLE SHOWING SUMMARY OF ADMISSIONS AND DISCHARGES.

| | | |
|---|---|---|
| Admissions ... | 180 | |
| Discharges ... | 131 | |
| Disease arrested, fit for suitable work ... | 49 | — 37.4 per cent. |
| Much improved ... | 33 | — 25.2 ,, |
| Improved ... | 30 | — 22.9 ,, |
| Not improved ... | 17 | |
| Died ... | 2 | |
| Total ... | 131 | |

The many difficulties of the early life of this Sanatorium have now been surmounted. The Sanatorium for consumptives in the three counties is now fully equipped and in full work, with its sixty beds occupied. We trust that the public will appreciate the advantages it has conferred on the district, and that its work will not be hampered by a long-standing debt on the building fund or a deficiency in its annual income.

